# Portable Microwave Radar Systems for Short-Range Localization and Life Tracking: A Review

**DOI:** 10.3390/s19051136

**Published:** 2019-03-06

**Authors:** Zhengyu Peng, Changzhi Li

**Affiliations:** 1 Aptiv PLC, Kokomo, IN 46902, USA; 2 Department of Electrical and Computer Engineering, Texas Tech University, Lubbock, TX 79409, USA; changzhi.li@ttu.edu

**Keywords:** portable radar, FMCW radar, MIMO, vital-Doppler, short-range localization, life tracking

## Abstract

Short-range localization and life tracking have been hot research topics in the fields of medical care, consumer electronics, driving assistance, and indoor robots/drones navigation. Among various sensors, microwave and mm-wave continuous-wave (CW) radar sensors are gaining more popularity in their intrinsic advantages such as simple architecture, easy system integration, high accuracy, relatively low cost, and penetration capability. This paper reviews the recent advances in CW radar systems for short-range localization and life tracking applications, including system improvement, signal processing, as well as the emerging applications integrated with machine learning.

## 1. Introduction

The first radars were developed as early as the 1930s [1]. Since then, radar systems have been widely used in military. Radar systems were only used in the military area due to their bulky sizes and high costs in the early days. Over recent decades, radar systems can be miniaturized and integrated onto a printed circuit board (PCB) thanks to the advance of high frequency integrated circuits (IC) and monolithic microwave integrated circuits (MMIC) [2,3,4]. With the advanced IC and packaging technologies, it is even possible to integrate a radar system into a single chip with antenna-on-chip (AoC) or antenna-in-package (AiP) technologies [5]. The operation frequencies of radar systems are going up to higher frequency bands. K-band (24 GHz) and W-band (77 GHz) have already been adopted in automotive radar systems [6]. Despite issues such as high path loss, higher operation frequency and smaller wavelength not only improving the sensitivity and resolution of radar systems, but also making radar systems further compact, radars’ applications have been extended from military to commercial areas. Researchers have been working on radar applications in through-wall detecting [7,8], indoor localization [9], driver assistance [6,10,11], and bio-medical applications [12,13,14,15], etc. Owing to the penetration capability of microwave signal, radar systems can detect human activities and vital signals behind obstacles, which makes it possible for earthquake rescues [16,17]. With the emerging of drones and robotic technologies over recent decades, short-range and indoor localization have also been a hot research topic [18,19,20,21,22,23]. Compared with camera-based localization technologies, which have some difficulties in getting accurate depth information of targets, radar systems can easily obtain targets’ ranges, speeds, and angles. Other solutions also have their own limitations compared with radar systems in short-range and indoor localization. For example, beacon-based solutions and inertial sensors require the targets to carry the devices all the time. In another relative short-range application, i.e., driver assistance, radar systems play important roles to prevent fatal crashes as well as minor accidents. In addition, with the arrival of autonomous vehicles, a radar system is one of the key sensors to make the autonomous driving technology possible. Moreover, a radar system is the only sensor that enable future autonomous vehicles operational at all the different weather conditions. Bio-medical is another important area for radar systems [12,13,14,15]. Radar systems have the capability to remotely detect and monitor tiny vital signs, such as respiration and heart rate. In contrast, current vital sign measurement devices and wearable technologies require the subject to have direct contact with the devices during the measurement, which affect the accuracy of the measurement results, especially in some applications such as sleep apnea monitoring [24]. With the ability to remotely detect vital signs and various mechanical movements, radar systems can also be used in various other emerging applications such as occupant detection [25,26], security monitoring [27,28], and gesture recognition [29,30,31,32].

Based on different types of waveforms, radar systems can be categorized into continuous-wave (CW) radar [33,34,35,36,37] and impulse radar [38,39]. Impulse radar has been used in various applications, such as vital signs detection, gesture recognition, and human tracking. Integrated chips of impulse radar systems are also available. On the other hand, CW radar has a simpler architecture, which allows easier system integration and lower power consumption. These features make CW radar attractive for mobile and portable applications. Various commercial CW radar chips and systems are available, especially for automotive applications. Table 1 lists several major manufacturers of commercial CW radar chips. Large semiconductor companies, such as Texas Instruments (Dallas, TX, USA), NXP (Eindhoven, The Netherlands), and Infineon (Neubiberg, Germany) have already released their solutions at 77 GHz for applications such as adaptive cruise control and autonomous driving. Table 2 shows several example manufacturers for commercial CW radar systems. Continental AG, Bosch, and Aptiv are well known tier 1 automotive suppliers, and have mature solutions for automotive front radar, rear radar and side radar. Recently, various startup companies, such as Arbe Robotics, Metawave, Caaresys, and Echodyne, are emerging. These companies are trying to apply innovative ideas to radar systems to enable radar systems in applications of new areas. For example, Metawave and Echodyne are using meta-materials in antenna arrays. Caaresys is developing radars to monitor the health conditions of drivers and passengers.

This review mainly focuses on recent advancements of CW radar systems in literature. CW radar systems can have different waveforms with different capabilities. Doppler radar or interferometry radar utilizes a single tone waveform to obtain motion information caused by the Doppler effect [3,40]. With relatively simple signal processing, a Doppler radar can provide superior accuracy in motion and displacement measurement [41,42,43,44]. Therefore, a Doppler radar is a very good option for applications such as vital sign detection [41,42,43], sleep monitoring, mechanical vibration detection, and structural health monitoring [45,46], etc., which do not require any range information. Frequency modulated continuous wave (FMCW) radar uses a modulated CW to sense both the range and Doppler properties of targets. FMCW radar is a better candidate for applications that require range information, such as localization, fall detection, life activity monitoring and gesture recognition. With the additional range information, it also helps to detect vital signs with the presence of multiple targets.

Generally, three main aspects are involved in the design of a radar system: (1) radar transceiver, (2) antenna or antenna array, and (3) radar signal processing. The radar transceiver includes the signal synthesizer, power amplifier (PA), low noise amplifier (LNA), mixer, and baseband circuitry, etc. Researchers and engineers are pushing the technology boundaries of every aspect in radar transceivers to achieve better performance with smaller size and lower cost. For different applicaitons, various radar architectures have been proposed and developed [35,36,37,47]. Antenna or antenna array is another important component in a radar system. For mobile and portable applications, patch antennas have been widely used. Antenna array has also been implemented to enable radar systems with 2D or 3D scanning capabilities. Researchers have been using phased array, digital beamforming and multiple-input multiple-output (MIMO) techniques to expand the capabilities of radar systems [36,37,48,49,50,51,52,53]. For radar signal processing, except for conventional methods, such as range-Doppler imaging [35] for FMCW radar and arc-tangent demodulation [54] for Doppler radar, researchers have been trying to use machine learning and an artificial neural network [27] to make radar systems smarter. This paper reviews the recent technical advance in portable FMCW radar systems for short-range localization and life tracking applications, including system hardware improvement, antenna array technologies and digital signal processing. Emerging applications will also be discussed.

This paper is organized as follows: Section 2 introduces the basic theory of FMCW radar and the conventional digital signal processing method to obtain targets’ information. Recent advancements in different aspects of radar systems will be reviewed in Section 3. Section 4 discusses the emerging applications. Conclusions are presented in Section 5.

## 2. Principle of FMCW Radar

This section introduces the basic principle of FMCW radar, antenna beamforming theory and radar signal processing methods.

### 2.1. FMCW Radar Transceiver

**Figure 1:**
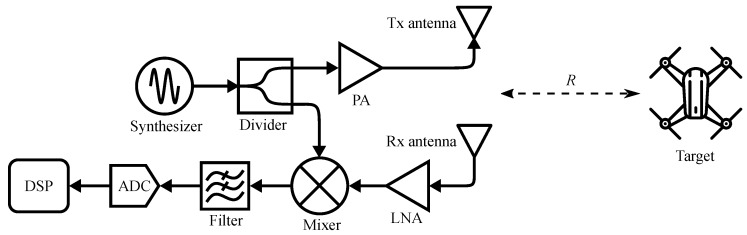
Top-level architecture of an FMCW radar system.

The principle of FMCW radar is illustrated in Figure 1. FMCW radar transmits a linear modulated RF signal, which is also referred as the chirp signal, to the target [55]. The reflected signal has a time delay Δτ, which is caused by the round-trip travelling distance of the electromagnetic wave between radar and the target:

(1) $$\Delta \tau =\frac{2R}{c},$$

where *R* is the distance between radar and the target, and *c* is the speed of light.

In the radar transceiver, the received signal $s_{R}\left(t\right)$ is multiplied with a replica of the transmitted signal $s_{T}\left(t\right)$ by an RF mixer. The output signal of the mixer is the radar baseband, which is also named as the beat signal $s_{b}\left(t\right)$. The beat signal can be expressed as [35]:

(2) $$s_{b}\left(t\right)=s_{T}\left(t\right)s_{R}^{*}\left(t\right)=A\exp\left(j\left(\frac{4\pi \gamma R(\tau )t}{c}+\frac{4\pi f_{c}R\left(\tau \right)}{c}+\Phi \right)\right),$$

where ∗ is the conjugation operator, *A* is the amplitude of the beat signal, R(τ) is the change of the target range versus time τ, and Φ is the negligible residual video phase (RVP), which can be disregarded. *t* is the “fast-time” and τ is the so-called “slow-time”:

(3) γ=B×CRF,

where *B* is the bandwidth of the chirp signal, and CRF is the chirp repetition frequency.

After performing Fourier transform of the beat signal, the frequency domain beat signal can be written as:

(4) $$S_{b}\left(f\right)=AT\exp\left(j\frac{4\pi f_{c}R\left(\tau \right)}{c}\right)\sin c\left(T\left(f-\frac{2\gamma R(\tau )}{c}\right)\right),$$

where *T* is the length of one chirp.

For Label (4), the peak of $S_{b}\left(f\right)$ is located at $f_{p}=2\gamma R\left(\tau \right)/c$. Thus, the range of the target can be obtained as:

(5) $$R\left(\tau \right)=\frac{f_{p}c}{2\gamma }=\frac{f_{p}c}{2B\times CRF}.$$

The other term in Label (4) shows the slow-time phase history ϕ(τ), which corresponds to the range evolution R(τ) of the target:

(6) $$\phi \left(\tau \right)=\frac{4\pi f_{c}R\left(\tau \right)}{c}.$$

As long as the coherence of the radar system is maintained, ϕ(τ) is only related to R(τ). Thus, the Doppler properties of the targets can be obtained by processing the beat signal along the slow-time τ.

### 2.2. Antenna Array and Beamforming

For an array of antennas, it is able to form different radiation patterns by controlling the phase and amplitude of the narrow band signal in each antenna channel. With this beamforming technique, FMCW radar will have an additional capability to discriminate the relative angles of targets. The phase and the amplitude of each antenna channel can be controlled either in the RF analog domain or the digital domain, which is referred to RF beamforming or digital beamforming, respectively [56].

**Figure 2:**
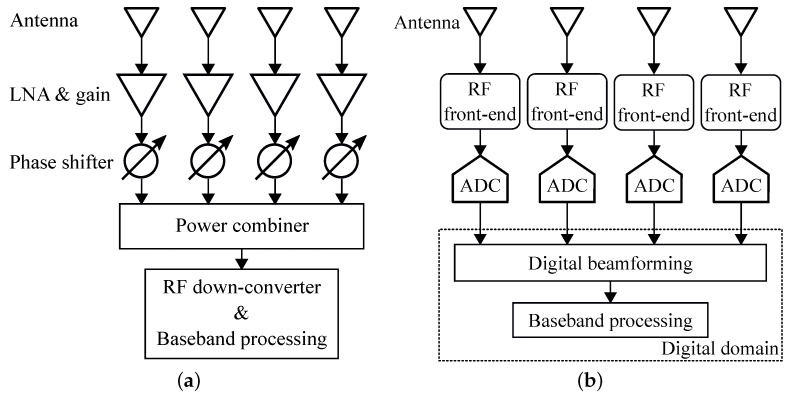
Block diagram of (**a**) RF beamforming architecture; (**b**) digital beamforming architecture.

The block diagram of an RF beamforming receiver is shown in Figure 2a. RF beamforming architecture usually doesn’t require high computational resources for beamforming processing. It also has relative lower power consumption compared with a digital beamforming architecture. However, an RF beamforming system suffers from beamforming accuracy due to phase errors from RF phase shifters. In addition, the current costs of high frequency, especially above K-band, phase shifters are very high. New RF beamforming techniques are emerging for higher frequencies, such as delay lines [57,58], LO phase-shifting [59,60] and vector sum [52,61,62]. On the other hand, with the advance of high-speed ADCs and DACs, as well as digital signal processors, digital beamforming systems have been widely used in wireless communication systems and high-end automotive radars. The block diagram of a digital beamforming receiver is illustrated in Figure 2b. Digital beamforming architecture has higher beamforming accuracy and more flexibility. However, due to the massive use of ADCs or DACs, it has a higher power consumption. Researchers have been looking for solutions to reduce the required number of ADCs and DACs for digital beamforming systems. Techniques such as sparse array [53,63], compressive sensing [36], path sharing [64,65,66,67] and MIMO [36,53] have been investigated to effectively reduce the hardware intensity of digital beamforming systems.

### 2.3. FMCW Radar Signal Processing

**Figure 3:**
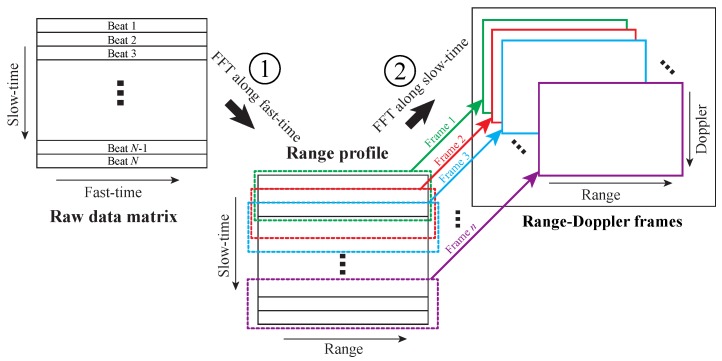
Signal processing procedure for an FMCW radar [34].

The basic signal processing for an FMCW radar includes two steps: (1) process “fast-time” to obtain range profile; (2) process “slow-time” to obtain moving properties of targets. Figure 3 shows the procedure of the two-step radar signal processing [34]. The raw data matrix consists of a series of time domain beat signals. The range profile can be obtained by applying Fourier transform along the “fast-time”. With the range profile, a series of range-Doppler frames can be calculated by windowing the range profile and performing Fourier transform along the “slow-time”. Range-Doppler frames reveals moving properties, as well as micro-Doppler properties of targets.

Advanced algorithms have been developed from the range profile and range-Doppler frames. For example, digital beamforming and angle-of-arrival (AoA) estimation algorithms can be applied to the range profile or range-Doppler frames to obtain angle information of targets. In addition, machine learning and artificial neural network have also been applied to radar signal processing.

## 3. Recent Advancements

Researchers have been working on different aspects of FMCW radar to make it ubiquitous for short-range localization and life tracking in our daily lives. In this section, recent advancements in radar architectures, RF beamforming techniques, MIMO implementation and life tracking algorithms for a portable FMCW radar will be reviewed and discussed.

### 3.1. Hybrid Radar

FMCW radar is one of the most popular CW radars, as it can easily and inexpensively obtain range information of targets. In addition, with coherent processing, Dopler information of targets can also be extracted with FMCW radar. However, the hardware and signal processing for an FMCW radar to obtain the Doppler information are much more complex than those of a Doppler radar. Moreover, a Doppler radar can easily reach sub-millimeter accuracy in displacement measurement, which, on the other hand, is quite challenging for an FMCW radar with limited bandwidth and processing resources [35]. To achieve a comparable accuracy, an FMCW radar requires a large transmit bandwidth and much more computational resources, which lead to an increase in system complexity and cost. A hybrid radar combines the advantages of both FMCW radar and Doppler radar. A hybrid radar can have high accuracy in displacement measurement without using a large bandwidth and complex signal processing. It can also obtain the absolute range information of targets by using a modulated waveform. Some modifications for a conventional CW radar hardware is necessary to realize a hybrid waveform.

**Figure 4:**
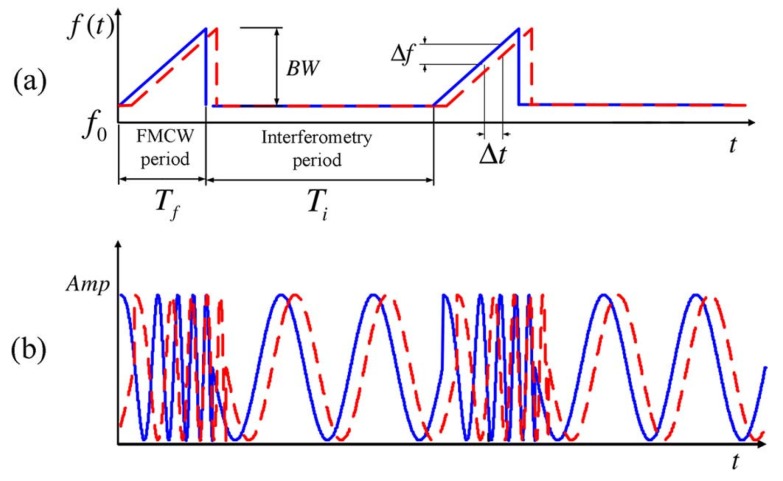
Transmitter (solid line) and receiver (dashed line) signals of the proposed hybrid system. (**a**) frequency domain; (**b**) time domain [47].

**Figure 5:**
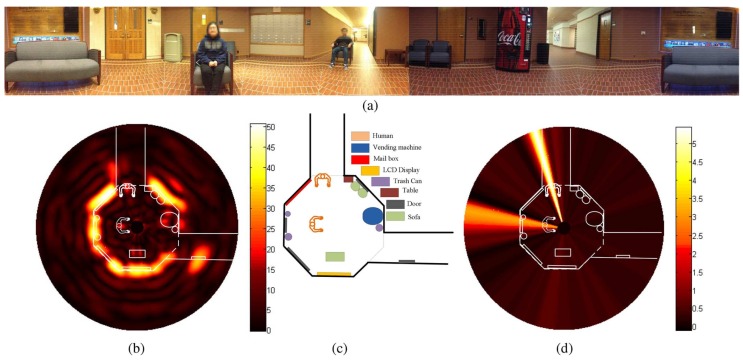
Experimental setup and results of the proposed hybrid radar system. (**a**) panorama photo of the environment; (**b**) 2D localization map with the FMCW mode; (**c**) room layout; (**d**) motion information with the Doppler mode [47].

Hybrid radar architectures have been proposed for 2D short-range localization in the literature [35,47]. The radar in [47] was built with National Instruments (NI) PXIe (Austin, TX, USA) and its transmitted signal is shown in Figure 4. With the proposed hybrid radar and a turntable, a 2D localization experiment has been carried out. Figure 5a is the panorama photo of the experiment environment. Figure 5b shows the 2D localization results obtained by the hybrid radar prototype with the FMCW mode. Figure 5c is the corresponded room layout in the experiment. The overlapped outline of the room and the detection results with the Doppler mode is illustrated in Figure 5d. The detection results matches very well with the actual layout of the room.

**Figure 6:**
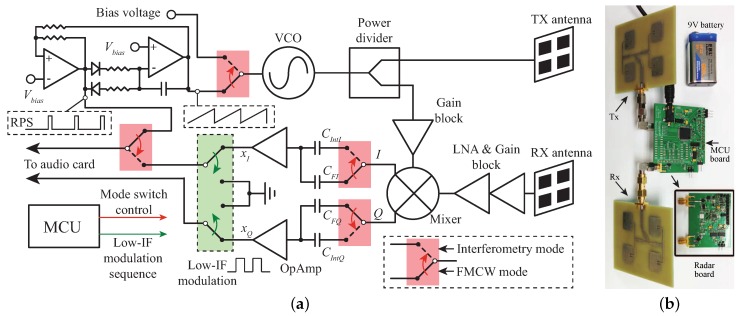
(**a**) simplifed schematic of the portable radar system; (**b**) photo of the radar prototype [35].

A portable hybrid radar was proposed and built in [35]. This 5.8 GHz hybrid radar was built with off-the-shelf components. Figure 6a shows the simplified schematic of the built 5.8 GHz portable hybrid radar prototype. In the FMCW mode, to minimize the size and cost of the system, a simple sawtooth generator circuit based on operational amplifiers is used to generate ramp voltage. The generated sawtooth range voltage is used to control a free-running voltage-controlled oscillator (VCO) to generate chirp signals [35]. The sawtooth generator circuit also simultaneously synthesizes the reference pulse sequence (RPS), which is synchronized to the sawtooth voltages. For the baseband, the audio card of a laptop is used to sample the analog baseband signal. On the other hand, in the Doppler mode, a low-intermediate-frequency (low-IF) modulation scheme was also used up-convert the baseband signal to an IF with simple analog switches. This low-IF modulation method makes it possible to sample the extreme low frequency vital signs with the audio card. A micro-controller is used to switch this radar prototype between the FMCW mode and the Doppler mode. The photo of the prototype is shown in Figure 6b.

**Figure 7:**
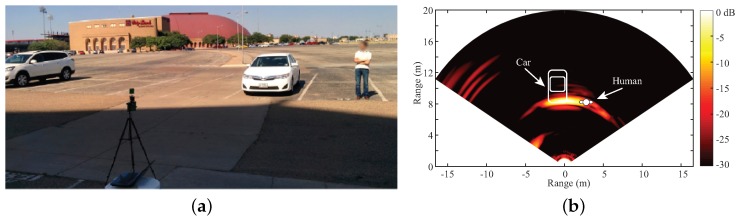
(**a**) 2D mapping experimental setup; (**b**) 2D mapping result [35].

Experiments have been taken with this portable hybrid radar prototype. Figure 7 represents the 2D localization result of a scene with a car and a human subject. Figure 7a is the photo of the experiment scene and Figure 7b shows the 2D localization result, which matches well with the layout of the scene. This radar prototype also shows the vital sign detection capability in [35].

### 3.2. Portable Radar with RF Beamforming

As have been discussed above, an RF beamforming system enables the FMCW radar with the beam scanning capability. However, a conventional phased array, especially at high frequencies, is expensive. Novel RF beamforming techniques are emerging for applications with higher frequencies. Researchers have designed complex delay lines and switch-antenna arrays to realize beam scanning for FMCW radar systems [57,58,68]. However, these methods usually have a limited number of steering angles. In addition, with the increased number of steering angles and antenna elements, the signal routing will be very difficult. The LO phase-shifting technique was introduced in [59], which designed a complex multi-phase VCO and multiple phase select switches to change different phases for LO. However, the steering angles and number of antenna elements are limited by the number of phase states for the multi-phase VCO.

**Figure 8:**
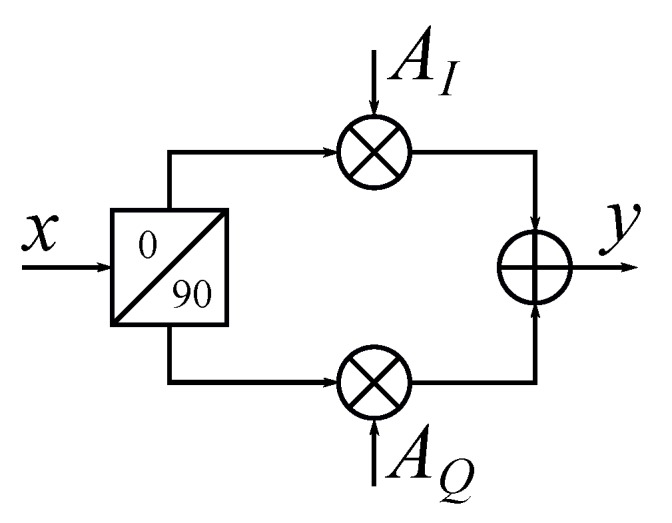
Conceptual diagram of a vector modulator [52].

Among these various approaches, the vector modulator technique, which is based on the concept of vector sum, is one of these optimal solutions for RF beamforming FMCW radar systems at higher frequencies [52,60,61,69]. The basic concept of a vector modulator is shown in Figure 8. Assume a single tone input signal $x=\sin\left(2\pi f_{c}t\right)$, where $f_{c}$ is the carrier frequency and *t* is time. In the vector modulator, the input signal is divided into an in-phase (*I*) signal and a quadrature (*Q*) signal by a quadrature power divider. The amplitudes of *I* and *Q* signals are controlled separately by a pair of variable gain amplifiers (VGA) or attenuators. Then, these two signals are combined together. The output signal *y* of the vector modulator can be expressed as:

(7) y=AIsin2πfct+AQcos2πfct=Re{Aejϕe−j2πfct},

(8) $$A=\sqrt{A_{I}^{2}+A_{Q}^{2}},$$

(9) $$\phi =\arctan\frac{A_{Q}}{A_{I}}.$$

It can be seen that the output signal of the vector modulator has the phase shift of ϕ and an amplitude change of *A* in Label (7). In addition, both ϕ and *A* can be controlled simultaneously through $A_{I}$ and $A_{Q}$. Based on the concept of vector modulator, an integrated phased-array chip working at 24 GHz has been developed by researchers in [61]. Vector modulators with VGAs have been used to realize the phase and amplitude control for a 22–24 GHz four-channel phase array in [61]. This CMOS phase array has the capability to achieve $360^{\circ }$ continuous phase shift for each channel as well as 0–20 dB amplitude tuning range.

**Figure 9:**
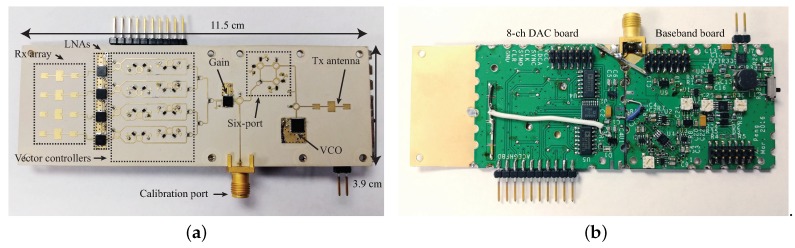
Photographs of the 24 GHz RF beamforming radar prototype. (**a**) top view; (**b**) bottom view [52].

**Figure 10:**
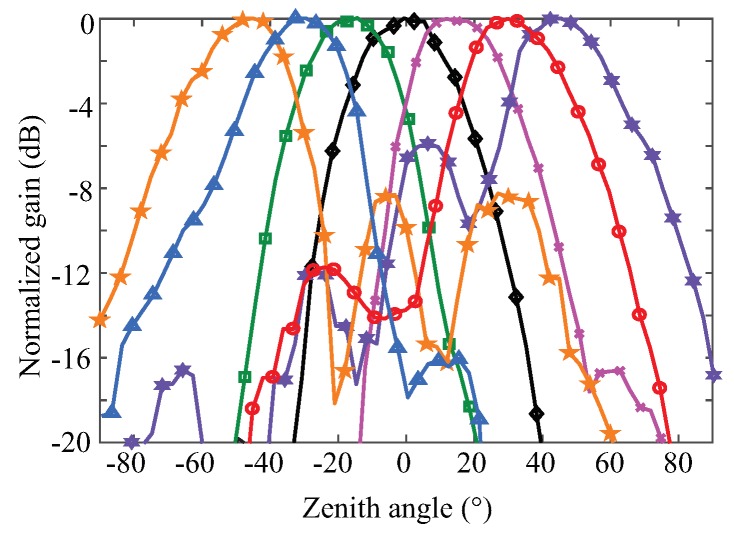
Measured radiation patterns of the array steering to $-45^{\circ }$, $-30^{\circ }$, $-15^{\circ }$, $0^{\circ }$, $15^{\circ }$, $30^{\circ }$, and $45^{\circ }$ at 23.75 GHz [52].

**Figure 11:**
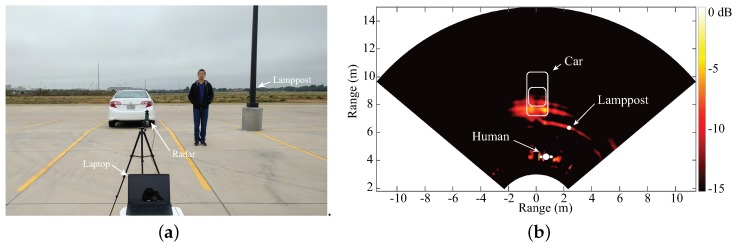
(**a**) experimental environment of short-range localization with the RF beamforming radar prototypes; (**b**) measured result of the 2D localization experiment [52].

Besides the achievements in integrated circuit design, a PCB realization of a K-band portable FMCW radar with RF beamforming array is also reported in [52]. Figure 9a,b are the photographs of the designed RF beamforming radar prototype. Both the transmitter and the receiver are included in the designed 24 GHz RF beamforming radar prototype. The transmitter uses a free-running VCO, controlled by a “sawtooth” voltage generator to synthesize the chirp signal, which covers the frequency band from 23.5–24 GHz. A signal patch antenna is used to transmit chirp signals. The receiver channel consists of a four-channel array, a six-port circuit and a baseband circuit. The main beam of the receiver array can be continuously steered with a range of $\pm 45^{\circ }$ on the H-plane by using a vector controller for each antenna channel. The vector controllers are realized with simple microwave structures and PIN diodes on a PCB. The audio card from a laptop is used to sample the beat signal of this FMCW radar. The measured receiver patterns when the antenna beam is tuned to different angles are plotted in Figure 10. With this portable RF beamforming FMCW radar, a 2D localization experiment has been performed. Figure 11a is the photo of the experimental scene. In this experiment, a car, a lamppost, and a human subject are in the field of view of the radar prototype. The scanning angle of the radar started from $-45^{\circ }$ to $45^{\circ }$ with a step size of 2.5°. The experiment results are illustrated in Figure 11b, where signatures of the car, the lamppost and the human subject can be clearly seen.

### 3.3. Sparse Array and MIMO

It is known that a digital beamforming system has high phase and amplitude accuracy compared with an RF beamforming system. However, it usually requires high-speed ADCs or DACs for all the antenna channels. Due to the massive use of high-speed ADCs or DACs, the cost and power consumption of a digital beamforming system are significantly increased. Researchers have been working on various approaches to reduce the hardware intensity of digital beamforming systems. One of these various approaches is using sparse array [53,63], which uses larger than half-wavelength antenna spacing to form the array. The sparse array increases the effective aperture of the array with fewer antenna elements. However, an equally-spaced sparse array has the issue of grating lobes, which introduces ghost targets for radar applications [56]. It is possible to remove the grating lobes of a sparse array by introducing some randomness in the antenna spacing [36,53]. The MIMO technique, which uses multiple transmitter channels and multiple receiver channels to synthesize a larger virtual array to improve the spatial resolution, is another effective approach that can significantly reduce the number of antenna channels [36,37,53]. In [36], a 77-GHz FMCW MIMO radar is proposed, which includes a linear non-uniformly spaced array and a SiGe single-chip FMCW radar transceiver. With the non-uniform MIMO array configuration, this radar is able to perform a 2D localization scan from $-90^{\circ }$ to $90^{\circ }$ without the issue of grating lobes.

**Figure 12:**
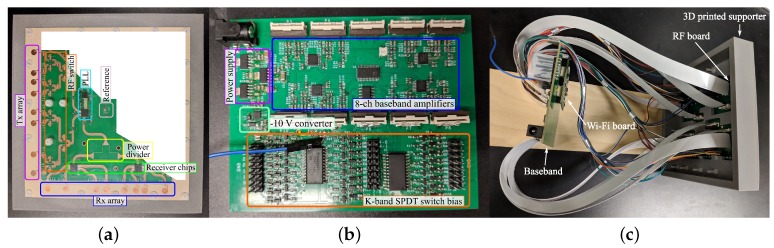
(**a**) RF board of the K-band 3D MIMO radar; (**b**) baseband board; (**c**) photo of the fully assembled 3D MIMO radar prototype [53].

To obtain a 3D localization capability, another 24-GHz FMCW MIMO radar is proposed in [53]. The photographs of this radar prototype are shown in Figure 12. Figure 12a is the photo of the RF board and Figure 12b is the photo of the baseband board. The photo of the fully assembled 3D MIMO radar prototype is shown in Figure 12c. An eight-element non-uniformly spaced array is optimized in this paper to achieve a narrow beam width, as well as removing the grating lobes. This optimized array configuration is then applied to both the transmitter array and receiver array with an “L” shape layout, as shown in Figure 12a. For the radar prototype, the RF board uses a K-band phase-locked loop (PLL) to synthesize the chirp signal for two transmitter channels and one LO channel. Customized K-band RF switches are used to extend the two-channel transmitters to eight channels. These eight-channel signals are transmitted by the transmitter array. On the receiver path, two four-channel radar receiver chips are used to process the received chirp signal. These two receiver chips have eight-channel baseband outputs in total, which are processed by eight baseband amplifiers. One of the eight channels is selected by an analog switch and sampled by the ADC on a Wi-Fi board. The sampled baseband data is transmitted to a computer for post-processing through Wi-Fi.

**Figure 13:**
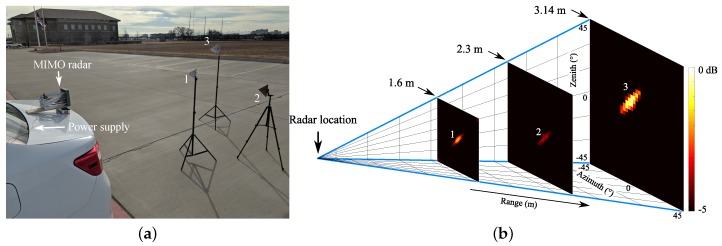
(**a**) photo of the experiment setup; (**b**) 3D localization mapping of the three targets with the portable MIMO radar prototype [53].

With the engineered MIMO radar prototype, an experiment with three targets with different ranges, heights and angles has been performed. The photo of the experimental environment is shown in Figure 13a. In the experiment, the radar prototype was fixed on the back of a car and the car battery was used to power the prototype. The targets were three corner reflectors, which were placed in front of the radar prototype with different ranges and heights. For a single scan, 64-channel beat signals were collected within about 0.64 s. The 3D localization result of the experiment is shown in Figure 13b after careful calibration. The detected ranges of the three corner reflectors are at 1.6 m, 2.3 m, and 3.14 m, respectively. The correct heights and azimuth angles are also obtained. For comparison, it needs a 12×12 planar array for a phased array to achieve the same performance of the designed MIMO radar. With a conventional MIMO radar without using a non-uniformly spaced array, it requires at least 12 transmitters and 12 receivers.

### 3.4. Life Tracking with Vital-Doppler

**Figure 14:**
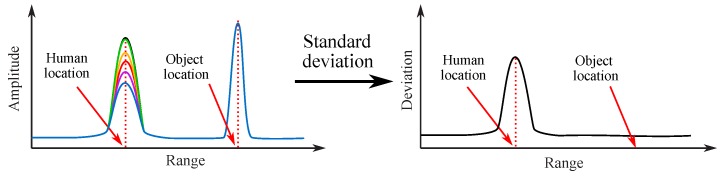
The vital-Doppler effect [35].

**Figure 15:**
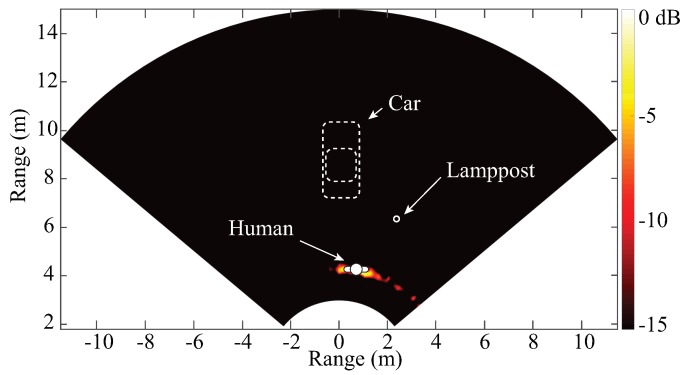
Extract the signature of a stationary living from Figure 11b [52].

It is quite easy for an FMCW radar to differentiate a moving target from stationary clutters. However, it is quite challenging to discriminate a stationary living target from other stationary objects. On the other hand, for living targets, such as human subjects, some special properties can be observed from the FMCW radar signal. When a human subject is standing or sitting still, his or her vital activities, such as respiration and heart beat, introduce significant variations in the amplitude of the reflected radar signal. This kind of effect is also referred to as the “vital-Doppler” effect [35]. Figure 14 shows the signal processing procedure life tracking with “vital-Doppler” effect. Firstly, multiple measurements are obtained for processing. Due to the “vital-Doppler” effect, the range profiles of a human target at different measurements have significant variations; however, the measured results for a stationary target are very stable. If the standard deviation of the range profiles of these different measurements is calculated, a peak will appear at the location of the human subject. Thus, the human subject can be discriminated from other stationary objects. This method has been applied in the experiment shown in Figure 11b to discriminate the human target. Figure 15 shows the 2D mapping results of human target discrimination with vital-Doppler effect. It can be clearly seen that the signatures of the stationary targets are minimized and the signature of the human subject is discriminated.

## 4. Emerging Applications

Various applications have been investigated with portable FMCW radar systems. In this section, several emerging applications will be introduced, including gesture sensing, fall detection, and human activity Categorizing.

### 4.1. Gesture Recognition

**Figure 16:**
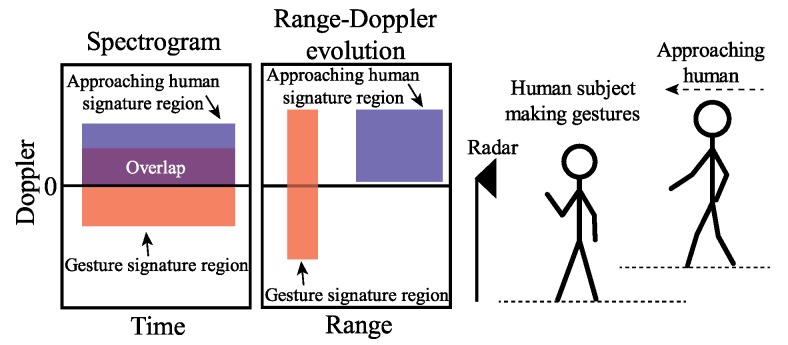
Illustration of the case when a human subject is making gestures while another human subject is approaching the radar [31].

**Figure 17:**
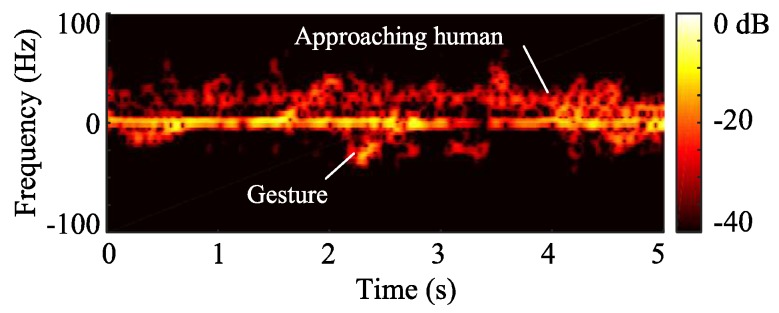
Spectrogram of two moving targets case shown in Figure 16 [31].

**Figure 18:**
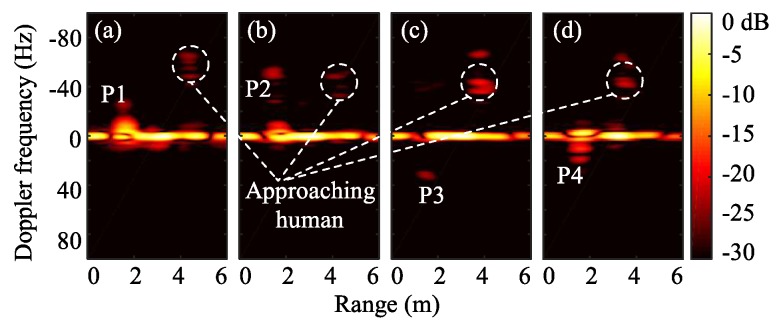
Range-Doppler frames of gesture recognition with the present to two moving targets at the same time. (**a**) Phase 1; (**b**) Phase 2; (**c**) Phase 3; (**d**) Phase 4 [31].

Cameras and complex image-processing algorithms have been used in advanced human–machine interface [70]. However, optical camera based solutions and their algorithms have a very high demanding for computational resources and are sensitive to ambient light. These two issues hinder the use of camera-based gesture recognition, especially with portable devices with limited computational capabilities. Doppler radar sensors show promising performance in detecting motion properties of targets [71]. Simple algorithms, such as time-frequency analysis, can be used in human gesture recognition. The advantages for a Doppler radar in gesture recognition include high integration, low demand for computational resources. However, one of the major issues for Doppler radar is that it is unable to separate motions from multiple moving targets. On the other hand, FMCW radar provides range information from targets, which help to separate motions from targets in different ranges. FMCW radar can have almost the same hardware architecture as Doppler radar, and its signal processing is a little more complex than that of a Doppler radar. However, compared with camera based solutions, FMCW radar still requires much less computational resources. Figure 16 illustrates a complex case when two targets are present in the field of view of an FMCW radar at the same time [31]. For the two targets, one human subject is making gestures, and, at the same time, the other person is walking toward the radar. With a Doppler radar with time-frequency analysis, the signatures of the two moving activities are overlapped, which makes it extremely difficult to separate. However, with an FMCW radar, it is straightforward to separate the two targets based on their different ranges. Then, the moving properties of the two different targets can be analyzed based on their micro-Doppler and micro-range signatures. The experiment has been performed with a portable 5.8 GHz Doppler radar prototype and a 5.8 GHz FMCW radar prototype. The experiment results match well with the analysis. The Doppler radar failed to separate the two motion signatures, shown in Figure 17, and the FMCW radar can successfully separate the two targets with different ranges and identify the gesture of one of the subjects by its micro-Doppler and micro-range features, shown in Figure 18.

Google’s project Soli [32] is one of the famous works for human gesture recognition with FMCW radar based on micro-Doppler features and machine learning. The Soli sensor was designed and developed at 60 GHz with AiP technology by Infineon Technologies (Neubiberg, Germany ). The Soli sensor has been demonstrated to be a promising technology for human–machine interaction interface.

### 4.2. Fall Detection

With a population aging, it has been a major concern in the society for the health of the seniors. An accidental fall is one of the most dangerous threats to the health of the seniors. To minimize the harm of a fall accident, it is essential to immediately alarm the family or care takers to give a necessary assist. Researchers have been working on different approaches to detect and identify fall accidents from other daily activities. These approaches include camera-based solutions [72,73,74], accelerometer-based solutions [75], and radar-based systems [76,77,78]. Techniques with accelerometers, as well as other wearable devices, require subjects to keep wearing sensors. The batteries of these kinds of these sensors need to be charged or replaced frequently. For camera-based solutions, complex algorithms, which require high computational resources, are used to extract motion features of human subject. However, these algorithms usually do not have sufficient accuracy in relatively complex environments. Moreover, camera-based solutions require the targets to be fully within the line of sight. Obstacles with even partial blockage can fail the detection for camera-based solutions. Doppler radar has also been used for fall detection by detecting micro-Doppler effects of fall accidents. Unlike wearable solutions, with Doppler radar, the subjects do not need to wear anything, which makes it more user-friendly. In addition, compared with camera-based solutions, Doppler radar does not have a high demand for computational resources, and it is able to penetrate obstacles. However, Doppler radar is not capable of isolating the fall incident with the presence of other moving activities.

**Figure 19:**
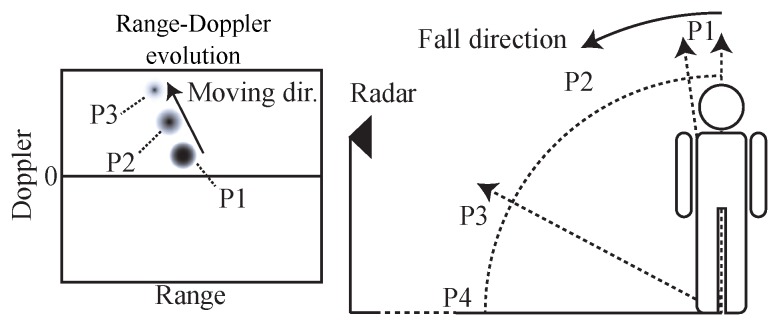
Illustration of a fall accident and the corresponding range-Doppler image evolutions [78].

**Figure 20:**
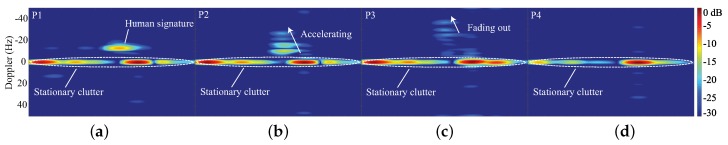
Frames of range-Doppler images during the fall incident. (**a**) P1; (**b**) P2; (**b**) P3; (**b**) P4 [78].

Fall detection with FMCW radar has been investigated in [78]. Besides the advantages of low requirements for computational resources and penetration capability, FMCW radar provides absolute range information of targets to help differentiate motion signatures from multiple targets. The Range-Doppler imaging method has been used to process the FMCW radar signal. Fall accidents can be distinguished from normal activities by using the properties of the adar cross-section (RCS), range, and Doppler on range-Doppler images during the movement of a subject. Figure 19 shows an example case when a human subject falls toward the radar. To simplify the analysis, this falling event is divided into four phases: P1, P2, P3, and P4, based on the motion properties. At P1, the subject starts to fall with a relatively low speed. Thus, the signature in range-Doppler image has low Doppler frequency. At P2, due to the acceleration, a sudden Doppler change can be observed for the signature of the human subject. When the human subject continues to fall, the RCS decreases due to the large tilt angle of the human subject at P3. Finally, the subject hits the ground, and the signature in range-Doppler image disappears. Experiment results shown in Figure 20 match well with the analysis of the four phases. The results of this research may enable FMCW radar based long-term remote fall detection applications without requiring the seniors to wear any sensing devices at home or nursing facilities.

### 4.3. Human Activity Categorizing

**Figure 21:**
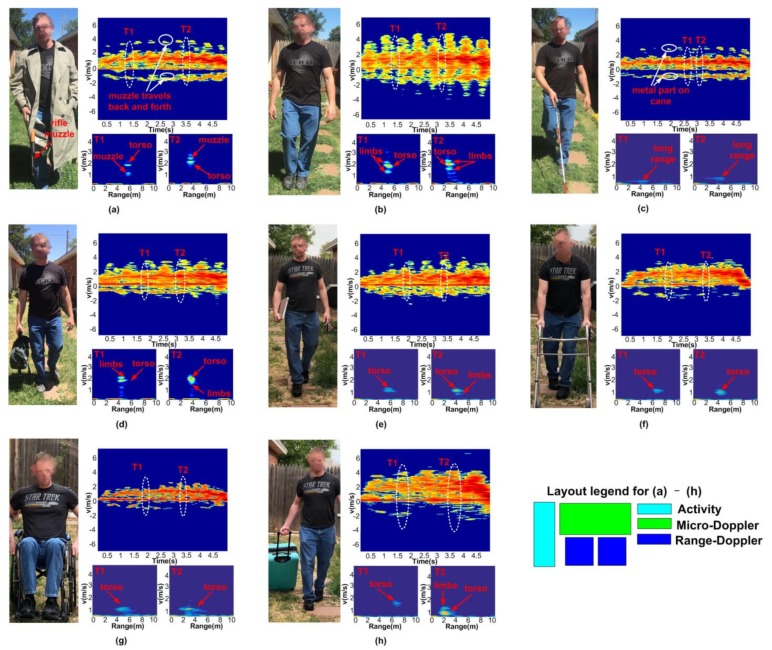
Micro-Doppler and range-Doppler properties of a human subject walking or moving at different conditions. (**a**) with a concealed rifle; (**b**) with natural arm swings; (**c**) with a cane for a blind person; (**d**) with holding a gym bag; (**e**) with carrying a laptop; (**f**) with a walker; (**g**) moving in a wheelchair; (**h**) with a rolling suitcase [27].

Micro-Doppler features have been used to differentiate targets among humans, animals and vehicles [79]. Researchers have found that it is possible to use micro-Doppler features to identify if the human subject is carrying a weapon [80]. According to the latest Federal Bureau of Investigation (FBI) report, the number of active shooting incidents is rising year by year. During 2014–2015, the total number of active shooting cases is six times higher than that during 2000–2001. Current technologies for detecting active shooters, such as acoustic gunshot identification and infrared camera gunfire flash detection, can only be triggered after a weapon is fired [81]. Although they can help to reduce the response time for the first responders, they can not prevent those incidents from happening. Thus, researchers are exploring radar based approaches to detect shooters armed with concealed rifle/shotgun and prevent active shooting incidents. Micro-Doppler and range-Doppler signatures have been used in active shooter detection in [27] with a portable radar system. In this work, the authors analyze the special gaits and gesture characteristics of a person walking with a concealed rifle. An ANN is adopted to classify a human subject walking with a concealed weapon, as well as seven other activities. Figure 21 shows the measured results and analysis of micro-Doppler and micro-range features among different activities. An average accuracy of 99.21% can be achieved in identifying these different activities shown in Figure 21.

## 5. Conclusions

Owing to the advancement of high frequency IC and MMIC, radar systems can be miniaturized and integrated onto a small PCB, which make radar technologies available for civil applications, such as short-range localization and life tracking. A portable FMCW radar has attracted significant attention from researchers across the world, and important improvements from different aspects of an FMCW radar system make it possible to use FMCW radar in various applications. Various hardware architectures have been proposed to expand the capabilities of FMCW radar in short-range localization and life-tracking applications. Advantage antenna and antenna array technologies have also been applied to FMCW radar to make it capable of 2D and 3D localization. To discriminate stationary living targets from other stationary clutters, a vital-Doppler effect with a statistic based algorithm is implemented. Various emerging applications with FMCW radar have been investigated, including gesture recognition, fall detection and active shooter detection with machine learning. It is expected that more advancements will be in FMCW radar to push the technology into more practical applications.

## Figures and Tables

**Table 1:** Commercial CW radar chips.

Manufacturer	Model No.	Frequency
Analog Devices	ADF4159, ADF5901, and ADF5904 chipset	24 GHz
Texas Instruments	AWR1642, AWR1443, etc.	77 GHz
NXP Semiconductors	TEF8181EN, TEF8102EN	77 GHz
Infineon	BGT24MTR11, BGT24LTR11N16, etc.	24 GHz
	RTN7735PL	77 GHz

**Table 2:** Commercial CW radar systems.

Manufacturer	Frequency	Example Model	Application
Continental AG	77 GHz	ARS441	Automotive
Bosch	77 GHz	LRR4	Automotive
Aptiv	77 GHz	ESR	Automotive
Arbe Robotics	-	-	Automotive
Metawave	77 GHz	-	Automotive
Caaresys	-	-	Automotive
Echodyne	-	ECHOGUARD	Industry
Innosent	24 GHz	ISYS-4004	Industry
RFBeam	24 GHz	K-LC1a V5	Industry
